# LAG-3 expression in microglia regulated by IFN-γ/STAT1 pathway and metalloproteases

**DOI:** 10.3389/fncel.2023.1308972

**Published:** 2023-11-08

**Authors:** Yuta Morisaki, Motoki Ohshima, Hikaru Suzuki, Hidemi Misawa

**Affiliations:** Division of Pharmacology, Faculty of Pharmacy, Keio University, Tokyo, Japan

**Keywords:** microglia, immune checkpoint molecule, LAG-3, IFN-γ, ADAM

## Abstract

Microglia are resident innate immune cells in the central nervous system (CNS) and play important roles in the development of CNS homeostasis. Excessive activation and neurotoxicity of microglia are observed in several CNS disorders, but the mechanisms regulating their activation remain unclear. Immune checkpoint molecules are expressed on activated immune cells and regulate their activation in peripheral immunity. However, the expression mechanism of immune checkpoint molecules in activated microglia is still unknown. Here, we analyzed the expression of immune checkpoint molecules in activated microglia using the mouse microglial cell line BV2 and primary cultured microglia. The expression of lymphocyte activation gene-3 (LAG-3), a type of immune checkpoint molecule, was increased in microglia activated by IFN-γ. IFN-γ-induced LAG-3 expression in microglia was suppressed by transfection of siRNA targeting STAT1. LAG-3 has two forms, membrane and soluble, and both forms were upregulated in microglia activated by IFN-γ. The production of soluble LAG-3 was suppressed by treatment with inhibitors of metalloproteinases such as ADAM10 and ADAM17. IFN-γ administration into cisterna magna of mice increased LAG-3 expression in spinal microglia. Furthermore, LAG-3 knockdown in microglia promoted nitric oxide production by IFN-γ. Our results demonstrate that LAG-3 expression in microglia is induced by the IFN-γ-STAT1 pathway and soluble LAG-3 production is regulated via cleavage of membranous LAG-3 by metalloproteinases including ADAM10 and ADAM17.

## 1. Introduction

Neuroinflammation, characterized by the activation of immune cells and the release of inflammatory molecules in the central nervous system (CNS), is a complex phenomenon that plays a pivotal role in neurological diseases (Hickman et al., 2018). Among the principal immune cells implicated in CNS inflammation, microglia are considered central to the orchestration of neuroinflammatory processes. Under normal physiological conditions, microglia exhibit a quiescent phenotype and constantly monitor their microenvironment (Haynes et al., 2006). However, in neurological disorders, aberrantly activated microglia are observed; these microglia continuously release inflammatory molecules, perpetuating a chronic inflammatory environment in the CNS, which leads to disease progression and the manifestation of neurological symptoms (Keren-Shaul et al., 2017). Given these detrimental effects, controlling microglial activation represents a possible therapeutic strategy for neurological diseases, but it remains unclear how microglial activation is regulated.

Immune cell activation is regulated by various mechanisms, such as activation-induced cell death, downregulation of stimulatory molecules and upregulation of inhibitory molecules (Frauwirth and Thompson, 2002; Brenner et al., 2008). Immune checkpoint molecules are key receptors expressed on activated immune cells that contribute to immune homeostasis by suppressing excessive immune and autoimmune responses (Paluch et al., 2018). For example, lymphocyte activation gene-3 (LAG-3), representative immune checkpoint molecule, has been reported to be expressed on T cells activated by various factors, governing cell proliferation and cytokine production (Huard et al., 1994). Immune checkpoints are a pivotal therapeutic target for cancer and autoimmunity: inhibiting immune checkpoints can augment anti-tumor immunity (Mehdizadeh et al., 2021) while enhancing immune checkpoint signaling can ameliorate autoimmunity (Burke et al., 2023). Thus, immune checkpoint molecules are crucial for maintaining the delicate balance between an appropriately protective immune response and excessive inflammation, and analyses of their expression mechanisms and functions are important for the understanding of immune cell activation.

Most of the research on immune checkpoint molecules has focused on cancer and peripheral immune cells such as T cells and NK cells (Buckle and Guillerey, 2021). In contrast, little is known about the role of immune checkpoint molecules in microglia, a key player as CNS-resident immune cells. In the present study, we evaluate the expression of immune checkpoint molecules in activated microglia and found that LAG-3 expression is induced by IFN-γ via STAT1 signaling. We found that soluble LAG-3 was produced via cleavage of membranous LAG-3 by metalloproteases such as ADAM10 and ADAM17 in microglia. We also demonstrated that nitric oxide (NO) production by IFN-γ was increased in LAG-3 knockdown microglia.

## 2. Materials and methods

### 2.1. Mice

Wild-type C57BL/6 mice were purchased from Sankyo Labo Service (Tokyo, Japan). All experiments were reviewed and approved by the Keio University Animal Care and Use Committee, and care was taken to minimize suffering and limit the number of animals used.

### 2.2. Antibodies and protease inhibitors

The following antibodies were used: rabbit anti-LAG-3 (1:1,000 for WB; ab209238, Abcam), mouse anti-LAG-3 (1:100 for ICC; MABF954, Merck Millipore), mouse anti-actin (1:3,000 for WB; MAB1501, Merck), rabbit anti-Iba1 (1:100 for ICC, 1:1,000 for IHC; 019-19741, WAKO), mouse anti-STAT1 (1:500; 661001, BioLegend), mouse anti-STAT1 Phospho (Ser727) Antibody (1:500; 686401, BioLegend). For flow cytometry analysis, Rat anti-mouse CD223 (LAG-3) PE (125208, BioLegend) and Rat IgG1, κ Isotype Ctrl Antibody PE (400408, BioLegend) were used. Following pharmacological protease inhibitors were used: GM6001 (10-1339, FOCUS Biomolecules), GI245023X (T7522, TargetMol), and TAPI-1 (18505, Cayman).

### 2.3. BV2 cell culture and transient transfection of siRNAs

The murine microglial cell line BV2 was cultured in RPMI supplemented with 10% FBS at 37°C under atmosphere 5% CO_2_/95% air. Cells were passaged at 60–70% confluence and the experiments were conducted using subconfluent cells in the proliferative phase at 60–80% confluence. BV2 cells were seeded in 6-well cell culture plate (3 × 10^5^ cells/well) for 24 h and then stimulated with the following reagents: 1 μg/ml lipopolysaccharide (LPS, Sigma), 100 U/ml interferon-gamma (IFN-γ, Peprotech), 25 ng/ml IL-4 (Peprotech), and 25 ng/ml IL-13 (Peprotech) in serum-free RPMI. After 24 h, the supernatants and whole cell lysates were collected for assays.

For transient transfection of siRNA, BV2 cells were seeded in 6-well or 24-well cell culture plate (3 × 10^5^ cells/well for 6-well, 7.5 × 10^4^ cells/well for 24-well) for 24 h. Using Lipofectamine 2000 (Thermo) according to the manufacturer’s instructions, the cells were then transfected with 100 nM siRNA targeting STAT1 (sc-44124, Santa Cruz Biotechnology) or LAG-3 (sc-44548, Santa Cruz Biotechnology). Scrambled control siRNA-A (sc-37007, Santa Cruz Biotechnology) was used as a control. Twenty-four hours after siRNA transfection, BV2 cells were cultured for another 24 h in a serum-free medium with or without 100 U/ml IFN-γ and used for assays.

### 2.4. Nitric oxide measurement

Nitric oxide concentration was measured by incubating cultured medium with an equal volume of Griess reagent (0.1% N-1-naphthyl-ethylene-diamine dihydrochloride, 1% sulfanilamide, and 2.5% phosphoric acid). After incubation for 20 min, the optical density was determined at a wavelength of 550 nm using a microplate reader Infinite 200 PRO (TECAN) with NaNO_2_ as a standard.

### 2.5. Cell surface biotinylation assay

BV2 cells were seeded in 6-well cell culture plate (3 × 10^5^ cells/well) for 24 h and then treated with IFN-γ or IL-13. After 24 h of stimulation, cells were incubated with 1.0 mg/ml sulfo-NHS-SS-biotin (Pierce) in PBS(+) for 45 min and solubilized with lysis buffer. Cell lysates were centrifuged at 21,600 × *g* for 10 min at 4°C. Biotinylated proteins were separated from non-biotinylated proteins by incubation with UltraLink Immobilized NeutrAvidin Protein Plus (Pierce) overnight at 4°C. The precipitated proteins were eluted with SDS sample buffer and analyzed.

### 2.6. Primary microglial culture

Cortexes from P0-2 C57BL/6 mice were stripped of meninges and blood vessels, minced with blades, dissociated with Trypsin and DNase I, cleared using a 70-μm cell strainer, and plated on poly-D-lysine-coated T25 flask in DMEM supplemented with 10% FBS, 100 U/ml penicillin and 100 μg/ml streptomycin. After 14 days to grow confluent mixed astrocyte/microglia population, microglia was isolated by mild-trypsin method (Saura et al., 2003). Trypsin–EDTA solution (0.25% trypsin, Thermo) diluted 1:3 in serum-free DMEM was applied to the mixed glial cells and the upper mixed glial cell layer was detached from the bottom of the flask after incubation with this mild trypsin for 20–30 min depending on the culture confluence at 37°C. Then trypsin was inhibited by adding DMEM supplemented with 10% FBS to the flask and the detached upper cells and the trypsin solution were discarded. All that were left at the bottom of the flask were adherent microglial cells and collected by adding 0.25% trypsin/1 mM EDTA solution. Collected primary microglia were seeded in 6-well culture plate at 1.2 × 10^6^ cells/well and used for assays.

### 2.7. Immunocytochemistry

BV2 cells were seeded onto pol-D-lysine-coated 9-mm ACLAR round coverslips. After stimulated cells were fixed with 4% paraformaldehyde in PBS(−), permeabilized with 0.1% TritonX-100 in PBS (PBS-T), blocked at room temperature with 3% normal donkey serum in PBS-T, and immunostained for 1 h at room temperature with the primary antibodies. The cells were then incubated with the appropriated fluorescence-labeled secondary antibodies (Thermo). After washing, coverslips were wet-mounted in Fluoromount-G (Southern Biotechnology). Immunostained cells were examined using Olympus FV-3000 confocal microscope system (Tokyo, Japan).

### 2.8. Real-time quantitative PCR

Total mRNA was extracted from cells using FastGene™ RNA Basic Kit according to the manufacturer’s instructions. Reverse transcription (1.5 μg of RNA) and quantitative PCR were performed with PrimeScript™ RT Master Mix (Takara Bio) and Taq Pro Universal SYBR qPCR Master Mix (Vazyme) using Thermal Cycler Dice Real Time System III (TP950; Takara Bio). PCR was performed using a two-step protocol. Initial denaturation of the cDNA at 95°C for 30 s was followed by 40 cycles of 95°C for 5 s and 60°C for 30 s, and a final dissociation stage entailing 95°C for 15 s, 60°C 30 for 30 s, and 95°C 15 s. Primer sequences are listed in Table 1. Relative gene expression normalized to mouse GAPDH was determined using the ΔΔCt method.

**TABLE 1:** Primer sequences used for quantitative real-time PCR.

Target description	Forward primer	Reverse primer
iNOS	CCCTTCAATGGTTGGT ACATGG	ACATTGATCTCCGTG ACAGCC
Arg1	AGGACAGCCTCGAG GAGGGG	CCTGGCGCGTGGCCAG AGATGC
LAG-3	CTCCATCACGTACAAC CTCAAGG	GGAGTCCACTTGGCAA TGAGCA
PD-1	CGTCCCTCAGTCAA GAGGAG	GTCCCTAGAAGTGC CCAACA
TIM-3	ACTGGTGACCCTCCA TAATAACA	ATTTTCCTCAGAGCG AATCCT
VISTA	AACAACGGTTCTAC GGGTCC	CGTGATGCTGTCAC TGTCCT
STAT1	GCCTCTCATTGTCACC GAAGAAC	TGGCTGACGTTGGAGA TCACCA
ADAM10	CACCAAAAACACCA GCGTGC	AGTGTCCCTCTTCATT CGTAGG
ADAM17	TACATAATACCCGGGTC ACACTC	AGTCTGCCTGGCTC ATCTTT
GAPDH	TGTGTCCGTCGTGG ATCTGA	TTGCTGTTGAAGTCG CAGGAG

### 2.9. Western blot

Cells were lysed in RIPA buffer (40 mM HEPES, pH 7.4, 150 mM NaCl, 10% glycerol, 1% Triton X-100, 0.5% DOC-Na, 0.1% SDS, 25 mM NaF, 1 mM NaV_3_O_4_) supplemented with 1× protease inhibitor cocktail (Nakarai Tesque). After centrifugation for 20 min at 26,000 × *g* and 4°C, aliquots of lysate containing 30 μg of protein were subjected to SDS-polyacrylamide gel electrophoresis, after which the proteins were transferred to polyvinylidene difluoride (PVDF) membranes (Immobilon-P; Merck Millipore). The membranes were then blocked for 30 min at room temperature in Tris-buffered saline (TBS; 10 mM Tris–HCl, pH 7.5 and 150 mM NaCl) containing 0.05% Tween-20 (TBS-T) with 5% non-fat skim milk or 1% BSA in TBS-T for detection of phosphorylated STAT1. Once blocked, the membranes were probed first with specific primary antibodies overnight at 4°C and then with the appropriate HRP-conjugated secondary antibodies (Bio-Rad). Blots were visualized using ECL Prime Western Blotting Detection Reagent (Cytiva) and were captured using an ODYSSEY Fc Imaging System (LI-COR Biosciences). For cell supernatant analysis, the medium was collected, concentrated using Amicon Ultra-0.5 3K and subjected to Western blot as above.

### 2.10. Flow cytometry analysis

BV2 cells were harvested by adding a solution of PBS containing 2 mM EDTA, 2% FBS, 20 mM HEPES, 100 U/ml penicillin and 100 μg/ml streptomycin. Cells were washed with PBS (no serum), then Zombie Violet™ Fixable Viability Kit (BioLegend) was added and incubated at room temperature for 15 min. After washing with PBS containing 2% FBS and 0.1% sodium azide and blocking Fc receptor by anti-CD16/CD32 antibody (BioLegend), cell surface labeling was performed for 30 min at 4°C. Flow cytometry analysis was performed using FACSAriaIII (BD Biosciences), and data were analyzed using FlowJo software (Tree Star).

### 2.11. Intracisternal injection

Mice were anesthetized by subcutaneous injection of MMB mixture (0.3 mg kg^–1^ medetomidine hydrochloride, 4 mg kg^–1^ midazolam, and 5 mg kg^–1^ butorphanol) and exposed the cisterna magna. Then, mice were secured in a stereotaxic frame and the head was tilted downward. A total of 400 μg/ml IFN-γ in 0.1% BSA/PBS was filled into PE10 tube (Polyethylene Tubing 0.61 mm OD × 0.28 mm ID) with a 30G needle, then the needle was inserted into cisterna magna. The needle was fixed in place by Aron Alpha^®^ A (Daiichi Sankyo) before injection. A total of 2.5 μl of IFN-γ or 0.1% BSA/PBS (as control) was injected at a rate of 1 μl/min using a syringe pump. To prevent backflow, the syringe was left in place for 5 min after injection. The tube was cut and sealed at its end.

### 2.12. Immunohistochemistry

Twenty-four hours after intracisternal injection, mice were anesthetized with isoflurane and then perfused transcardially with PBS(−), followed by 4% paraformaldehyde (PFA) in 0.1 M phosphate buffer at pH 7.4 (PB). Cervical spinal cords were sampled and postfixed in the same fixative overnight at 4°C, after which it was immersed in 20% sucrose in PB overnight at 4°C, then the tissue was frozen in OCT compound (Sakura Finetek). Sections were sliced at 40 μm using a cryostat, and free-floating sections were immunohistochemically processed as previously described (Morisaki et al., 2016). For confocal scanning fluorescence microscopy, sections were incubated with primary antibodies and then with Alexa Fluor-conjugated secondary antibodies (Thermo) and wet-mounted in Fluoromount-G. Images were obtained by using Olympus FV-3000 confocal microscope system (Tokyo, Japan). Quantitative analyses of imaging measurement were performed using Fiji (ImageJ) and three spinal cord sections per animal were used.

### 2.13. Statistical analysis

All data are presented as the mean ± SEM. Statistical analyses were carried out using GraphPad Prism 10 software. Statistical significance was tested with Student’s *t*-test, one-way ANOVA with Dunnett’s *post-hoc* test or two-way ANOVA with Tukey’s multiple comparisons test. Differences were considered statistically significant at *p* < 0.05.

## 3. Results

### 3.1. mRNA expression of immune checkpoint molecules in activated microglia

**FIGURE 1:**
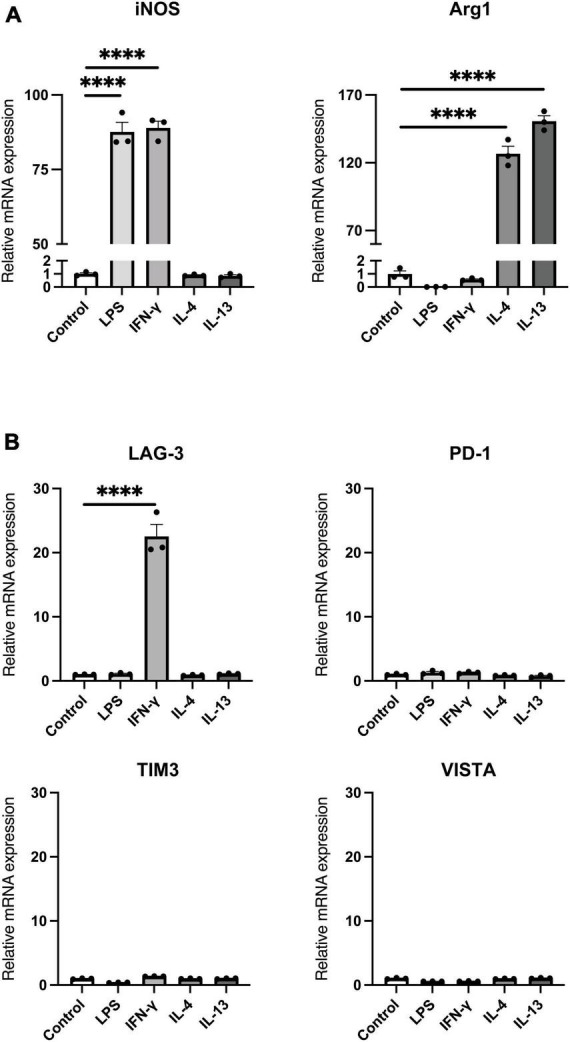
mRNA expression of immune checkpoint molecules in activated microglia. BV2 microglia cells were treated for 24 h with indicated stimulants, after which each mRNA levels were measured using quantitative real-time PCR. **(A)** mRNA expression of pro-inflammatory/anti-inflammatory activation markers. **(B)** Immune checkpoint molecule mRNA expression in activated microglia. Statistical analyses were performed using one-way ANOVA, followed by Dunnett’s *post-hoc* test; ****p < 0.0001. Error bars represent SEM.

Activated microglia are classified as neurotoxic pro-inflammatory microglia or neuroprotective anti-inflammatory microglia, depending on the differences in releasing inflammatory/anti-inflammatory mediators (Ransohoff, 2016; Tang and Le, 2016). We first investigated mRNA expression of immune checkpoint molecules in BV2 microglial cells polarized to pro-inflammatory or anti-inflammatory activated state. Stimulation of BV2 cells with LPS or IFN-γ increased mRNA expression of iNOS, indicating a polarity shift toward pro-inflammatory state. On the other hand, stimulation with IL-4 or IL-13 increased mRNA expression of Arg1, confirming a polarity shift toward anti-inflammatory state (Figure 1A). Under these conditions, we found that only IFN-γ increased LAG-3 mRNA expression (Figure 1B).

### 3.2. IFN-γ increases both membrane-bound and soluble forms of LAG-3 in microglia

**FIGURE 2:**
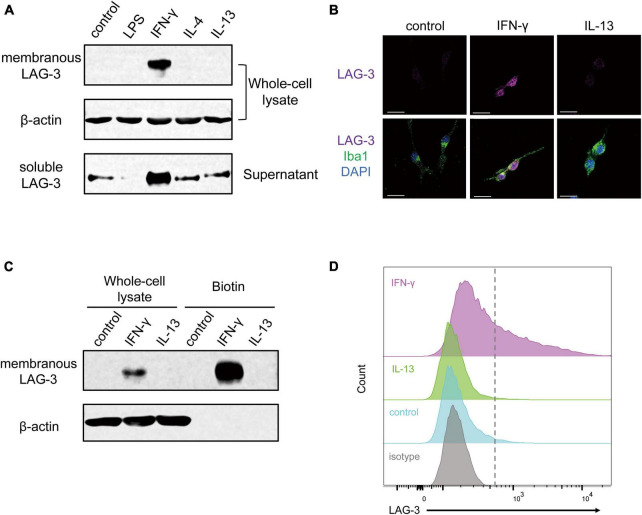
IFN-γ induces membranous and soluble LAG-3 expression in microglia. **(A)** LAG-3 expression was analyzed by Western blotting in stimulated BV2 cells. **(B)** Representative images of LAG-3 expression in stimulated BV2 cells. Cells were immunofluorescent labeled with antibodies against LAG-3 or Iba1. Nuclei were stained by DAPI (scale bar, 20 μm). **(C)** Detection of LAG-3 at the plasma membrane by cell surface biotinylation assay. **(D)** Histogram showing the fluorescent intensity of cell surface LAG-3 in IFN-γ- or IL-13-stimulated BV2 cells by flow cytometry. The images shown are representative of three independent experiments.

Next, we assessed the protein level of LAG-3 after each stimulation by Western blot. Since LAG-3 has two forms, membranous LAG-3 and soluble LAG-3 (mLAG-3 and sLAG-3) (Li et al., 2004), we analyzed both cell lysates and cell culture supernatants. Similar to the gene expression data, mLAG-3 was increased in BV2 cell lysates stimulated with IFN-γ, and an increase of sLAG-3 was also observed in the supernatant (Figure 2A). We also performed immunocytochemical staining to evaluate the expression of LAG-3 in stimulated BV2 cells. Consistent with Western blotting, immunocytochemical staining demonstrated the upregulation of LAG-3 in IFN-γ-stimulated BV2 cells (Figure 2B). It has been reported in other immune cells that LAG-3 expressed upon stimulation requires trafficking to the cell surface to exert its function (Bae et al., 2014). We examined whether mLAG-3, whose expression was upregulated by IFN-γ, existed on the plasma membrane surface. Cell surface biotinylation assay and flow cytometry indicated that mLAG-3 is present on the plasma membrane surface after stimulation by IFN-γ (Figures 2C, D).

### 3.3. LAG-3 expression of microglia is under control of the IFN-γ-STAT1 pathway

**FIGURE 3:**
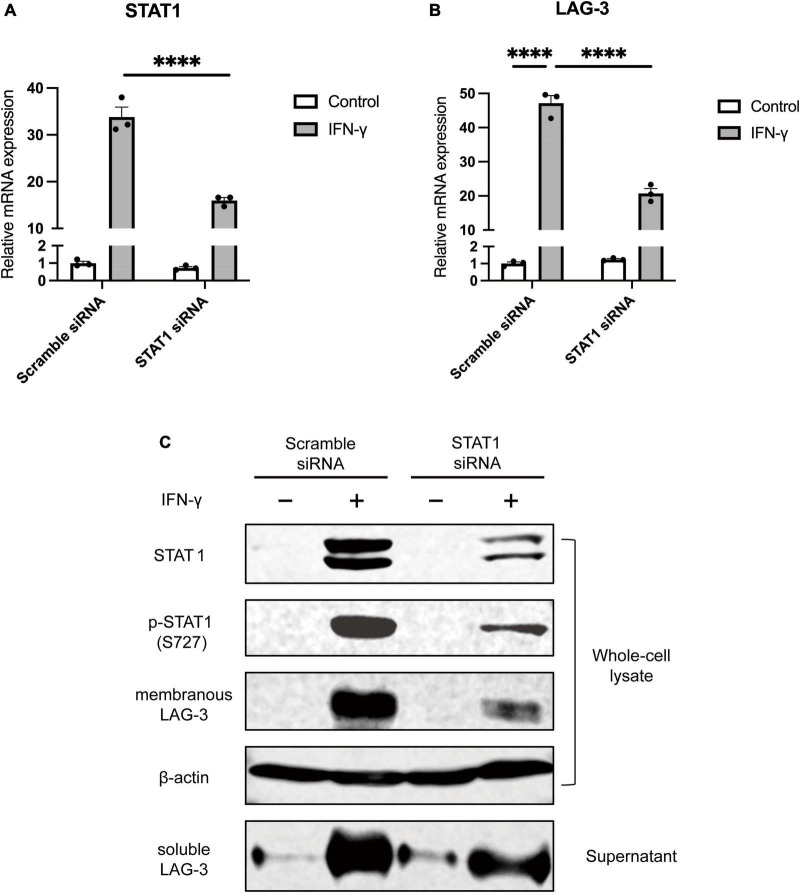
STAT1 knockdown suppresses IFN-γ-induced LAG-3 expression in microglia. BV2 cells were transfected with scramble control or STAT1 siRNA. After incubation with the siRNAs, IFN-γ was added and the cells were harvested. **(A)** STAT1 mRNA level was measured using quantitative real-time PCR to confirm the efficiency of knockdown by siRNA. **(B)** LAG-3 mRNA was measured in the siRNA-treated BV2 cells. **(C)** LAG-3 expression in siRNA treated BV2 cells was analyzed by Western blotting. The image shown is representative of three independent experiments. Statistical analyses were performed using two-way ANOVA, followed by Tukey–Kramer *post-hoc* test; ****p < 0.0001. Error bars represent SEM.

IFN-γ regulates gene expression via the JAK-STAT1 pathway, and phosphorylated STAT1 by activated JAK plays a critical role in inducing the transcription of specific genes (Hu et al., 2021). To investigate the signal transduction pathway leading from IFN-γ stimulation to the increase in LAG-3 transcription, BV2 cells were transfected with siRNA targeting STAT1. BV2 cells stimulated with IFN-γ showed an increase in STAT1 mRNA, while transfection with STAT1 siRNA suppressed the increase in STAT1 mRNA upon IFN-γ stimulation (Figure 3A). Expression of LAG-3 mRNA by IFN-γ was suppressed in STAT1 knockdown BV2 cells compared to that in BV2 cells transfected with scramble siRNA (Figure 3B). Consistent with the gene expression data, Western blot analyses suggested that STAT1 knockdown in BV2 cells suppressed IFN-γ-induced expression of un-phosphorylated and phosphorylated STAT1 protein, along with suppression of mLAG-3 in cell lysates and sLAG-3 in cell culture supernatants (Figure 3C). These results suggest that LAG-3 expression is under the control of IFN-γ-STAT1 axis in microglia.

### 3.4. Soluble LAG-3 is cleaved from membranous LAG-3 by metalloproteinases including ADAM10 and ADAM17

**FIGURE 4:**
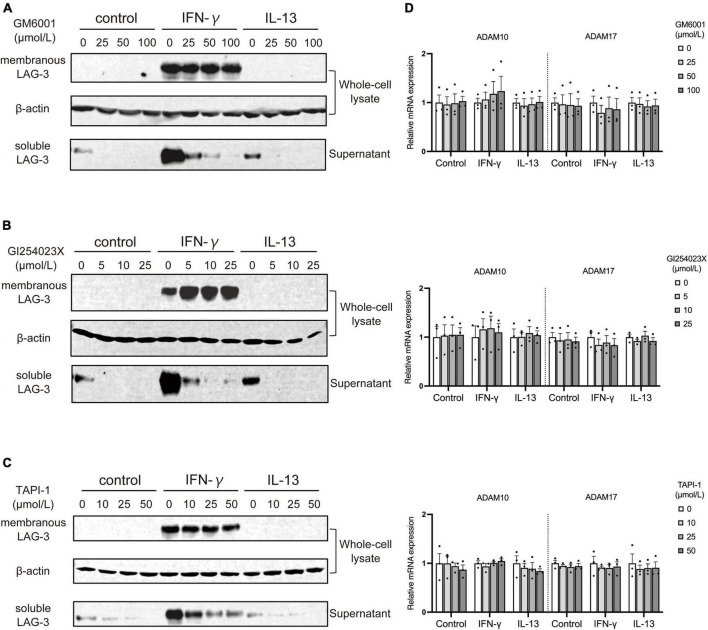
Metalloproteases including ADAM10 and ADAM17 cleave membranous LAG-3 and produce soluble LAG-3 in microglia. BV2 cells were treated by indicated stimulants in the presence of following metalloproteinase inhibitors, after which cell lysate and culture supernatant were analyzed by Western blotting; **(A)** pan-metalloproteinase inhibitor GM6001, **(B)** ADAM10 inhibitor GI254023X, **(C)** ADAM17 inhibitor TAPI-1. Representative blots out of three experiments. **(D)** ADAM10 and ADAM17 mRNA expression was not changed in the presence of each inhibitor. Error bars represent SEM.

As described above, microglia activated by IFN-γ showed increased expression of sLAG-3 in addition to mLAG-3, but the mechanism of sLAG-3 production in microglia is not clear. It has been reported that mLAG-3 expressed on activated T cells via TCR signaling is cleaved by metalloproteinases and released as sLAG-3 (Li et al., 2007). Therefore, we investigated whether metalloproteinases are also involved in the production of sLAG-3 in microglia by using GM6001, a pan-metalloproteinase inhibitor (Ethell et al., 2002). The result showed that GM6001 inhibited the production of sLAG-3 in IFN-γ-treated BV2 cells in a dose-dependent manner (Figure 4A), suggesting that metalloproteinases are involved in the production of sLAG-3 in microglia. Next, we focused on the ADAM family of metalloproteases, ADAM10 and ADAM17, which are known to be important for cell surface cleavage of immune-related transmembrane proteins (Lambrecht et al., 2018). Using selective inhibitors (ADAM10 inhibitor: GI254023X, ADAM17 inhibitor: TAPI-1), we investigated the production of sLAG-3 in BV2 cells upon IFN-γ treatment. Both inhibitors suppressed the contents of sLAG-3 in cell culture supernatant (Figures 4B, C). These inhibitors had no effect on ADAM10 and ADAM17 mRNA expression in BV2 cells (Figure 4D), suggesting that the production of sLAG-3 in microglia is due to cleavage by the metalloproteinases including ADAM10 and ADAM17.

### 3.5. IFN-γ induces LAG-3 expression in primary microglia and *in vivo*

**FIGURE 5:**
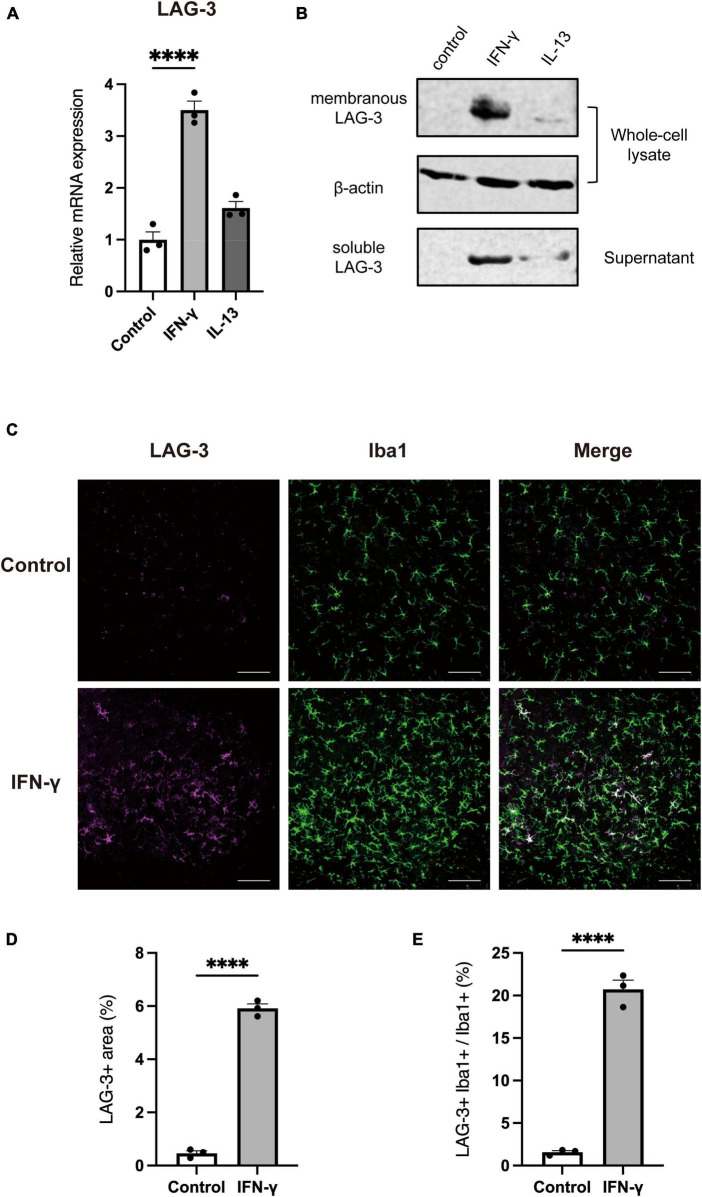
IFN-γ induces LAG-3 expression in primary cultured microglia and *in vivo*. LAG-3 expression was analyzed in stimulated primary microglia by **(A)** quantitative real-time PCR and **(B)** Western blotting. **(C)** Representative image of LAG-3 expression in mouse cervical spinal cord after intracisternal injection of IFN-γ (scale bar, 100 μm). **(D,E)** Quantification of LAG-3^+^ area and LAG-3^+^Iba1^+^/Iba1^+^ area (*n* = 3). Statistical analyses were performed using Student’s *t*-test or one-way ANOVA, followed by Dunnett’s *post-hoc* test; ****p < 0.0001. Error bars represent SEM.

Next, we analyzed whether IFN-γ induces LAG-3 expression in primary microglia and *in vivo*. Primary microglia were cultured in the presence of IFN-γ and analyzed for LAG-3 expression. Similar to the above analyses in BV2 cells, LAG-3 mRNA expression was upregulated (Figure 5A) and Western blot analysis also revealed an increased expression of mLAG-3 in cell lysate and sLAG-3 in the supernatant in IFN-γ treated primary microglia (Figure 5B). Furthermore, we analyzed the expression of LAG-3 in the cervical spinal cord of mice after injecting IFN-γ into cisterna magna. We found that the IFN-γ injection induced upregulation of LAG-3 in the spinal cord and LAG-3 expression in Iba1+ microglia (Figures 5C–E). These results indicate that IFN-γ also induced LAG-3 expression in primary cultured microglia and *in vivo*.

### 3.6. LAG-3 knockdown promotes IFN-γ-induced NO production in microglia

**FIGURE 6:**
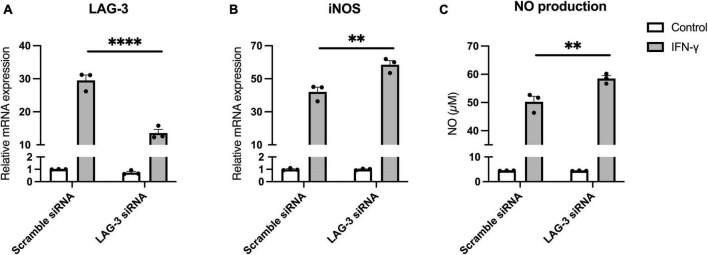
Lymphocyte activation gene-3 (LAG-3) knockdown promotes inflammatory activation by IFN-γ. BV2 cells were transfected with scramble control or LAG-3 siRNA. After incubation with the siRNAs, IFN-γ was added and the cells were harvested. **(A)** LAG-3 mRNA level was measured using quantitative real-time PCR to confirm the efficiency of knockdown by siRNA. **(B)** iNOS mRNA was measured in the siRNA-treated BV2 cells. **(C)** NO concentration was determined in culture supernatants of the siRNA-treated BV2 cells. Statistical analyses were performed using two-way ANOVA, followed by Tukey–Kramer *post-hoc* test; **p < 0.01, ****p < 0.0001. Error bars represent SEM.

Finally, to investigate the functional significance of LAG-3 expressed in IFN-γ-activated microglia, we transfected BV2 cells with siRNA targeting LAG-3 and examined their response to IFN-γ stimulation. In BV2 cells transfected with LAG-3 siRNA, we confirmed that LAG-3 mRNA expression by IFN-γ was suppressed (Figure 6A). Expression of iNOS mRNA by IFN-γ was increased in LAG-3 knockdown BV2 cells compared to that in BV2 cells with control siRNA (Figure 6B). Furthermore, we measured NO, an inflammatory mediator, in cell culture supernatants and found that IFN-γ-induced NO production was increased in LAG-3 knockdown BV2 cells, consistent with an increased expression of iNOS mRNA (Figure 6C). These results indicate that LAG-3 knockdown increased NO production by IFN-γ in microglia.

## 4. Discussion

Activated immune cells regulate their activation states by expressing immune checkpoint molecules, but its expression mechanism in activated microglia has not been clarified. In this study, we analyzed the expression of immune checkpoint molecules in microglia activated by various stimuli. We found that IFN-γ-induced stimulation increased the expression of both mLAG-3 and sLAG-3 in BV2 microglial cells. We demonstrated that STAT1 is involved in the signal transduction leading to LAG-3 induction by IFN-γ. The cleavage of mLAG-3 by metalloproteinases including ADAM10 and ADAM17 was shown to be involved in the production of sLAG-3 in microglia. We also showed that NO production by IFN-γ was increased in LAG-3 knockdown microglia. To our knowledge, this is the first study to examine the expression mechanism and functional significance of LAG-3 in microglia.

A recent report demonstrated that LAG-3 expression is elevated in hippocampal microglia of patients with major depression and that microglial LAG-3 is involved in the antidepressant effects of electroconvulsive therapy (Rimmerman et al., 2022). It has also been reported that the expression of LAG-3 is associated with activated microglia in the hippocampus of patients with bipolar disorder (Naggan et al., 2023). Although these reports do not provide detailed mechanisms of LAG-3 expression in microglia, the formation of an inflammatory environment and increased inflammatory cytokines, especially IFN-γ, have also been reported in such diseases (Gabbay et al., 2009). Our results suggest that the increased expression of LAG-3 observed in these diseases could be also IFN-γ mediated.

To date, the majority of studies on LAG-3 have focused on T cells (Burnell et al., 2022). LAG-3 is expressed on T cells through T cell receptor (TCR) signaling and cytokine stimulation. In T cells, it is generally understood that LAG-3 negatively regulates immune responses via suppression of proliferation and production of cytotoxic factors and cytokines (Durham et al., 2014). In the present study, among several microglial activation stimuli, only IFN-γ induced the expression of LAG-3. IFN-γ can alter microglial reactivity and enhance glial responses to other cytokines or pathogens. For example, simultaneous exposure to IFN-γ and TNF-α significantly increases NO production in microglia compared to exposure to IFN-γ alone (Mir et al., 2008). Furthermore, in our results, LAG-3 knockdown promoted IFN-γ-induced NO production in microglia. Thus, LAG-3 possibly acts as a negative feedback machinery that suppresses IFN-γ-dependent activation in microglia. Previous studies have identified MHC class II molecules (MHC-II), fibrinogen-like protein 1 (FGL-1), and Galectin-3 (Gal-3) as ligands that can bind to LAG-3 (Aggarwal et al., 2023). Focusing on the CNS, MHC-II, and Gal-3 have been reported to be expressed in activated microglia, and Gal-3 in particular contributes to increased production of inflammatory mediators in microglia (Burguillos et al., 2015). To further elucidate the function of LAG-3 in activated microglia, it is necessary to investigate the association of these molecules with LAG-3 expressed in microglia in the future.

In addition to mLAG-3, sLAG-3 was also increased by IFN-γ treatment in microglia. sLAG-3 has been reported to be produced in activated T cells by cleavage of mLAG-3 by metalloproteinases (Li et al., 2007). We confirmed that similar to T cells, the production of sLAG-3 in microglia is responsible for metalloproteinases including ADAM10 and ADAM17. Although the detailed physiological roles of sLAG-3 are largely unknown, previous studies suggest that sLAG-3 promotes Th1 cell responses by stimulating T cell proliferation through induction of dendritic cell activation and maturation (Andreae et al., 2002). Within other immune checkpoint molecules, soluble forms of PD-1 have also been suggested to regulate immune cell function by binding to the ligand PD-L1 and inhibiting the membrane PD-1/PD-L1 axis (Khan et al., 2020). Therefore, sLAG-3 produced by microglia may bind to the ligands and function as a decoy or functional blocker, contributing to the regulation of the immune environment in the CNS. Increased production of IFN-γ and microglial activation are observed in CNS-related diseases such as stroke and spinal cord injury (Tsuda et al., 2009; Zhang et al., 2020). Thus, elucidating the expression levels and functions of sLAG-3 could provide insight into the pathophysiology of such diseases.

## 5. Conclusion

In summary, we found that microglia activated by IFN-γ express LAG-3 via STAT1 pathway. We also demonstrated that sLAG-3 is produced in microglia via cleavage of mLAG-3 by ADAM10 and ADAM17 metalloproteinases. In addition, we proposed the possibility that LAG-3 is involved in the regulation of activated microglia. Our results may have implications for the involvement of LAG-3 in several diseases involving microglial activation.

## Data availability statement

The raw data supporting the conclusions of this article will be made available by the authors, without undue reservation.

## Ethics statement

The animal study was approved by the Keio University Animal Care and Use Committee. The study was conducted in accordance with the local legislation and institutional requirements.

## Author contributions

YM: Conceptualization, Investigation, Validation, Writing – original draft, Writing – review and editing. MO: Investigation, Writing – review and editing. HS: Investigation, Writing – review and editing. HM: Supervision, Writing – review and editing.
